# Supplementary material for: Mouse CCL9 Chemokine Acts as Tumor Suppressor in a Murine Model of Colon Cancer
Source: Curr Issues Mol Biol. 2023 Apr 15;45(4):3446–61. doi: 10.3390/cimb45040226 (PMC10136558; doi:10.3390/cimb45040226)
Supplement: Supplementary file 1 [file cimb-45-00226-s001.zip › cimb-2284063-supplementary.pdf]

Supplementary analysis

Our bioinformatics analysis of our own microarray data and public data repositories suggests a close link between CCL9 and T cell receptor (TCR) genes expressed by RORγt + T cells. In our microarray analysis, we observed upregulation of multiple genes related to TCR construction and function, such as Traj40, Trbv13-2, Traj39, Traj45, Trav9n-4, Trav9d-1, Trbv30, Trav9-1, and Trav7d-3 (figure 1A). As expected, mining Traj40 expression among different cells shows high specificity toward expression in T cells (Figure 1B). Interestingly, our analysis also showed that Rorc, the main transcription factor of Th17 and Tc17, is upregulated (Figure 1C). FoxP3 expression is downregulated, and similarly, Gata3 is not significantly differentiated. Thus, these findings exclude Tregs and CD4+ Th2 from significant potential positive regulation by CCL9. Next, 2e analyzed a single-cell RNA-seq dataset for human colorectal cancer (GSE222300) (Figures 1D, 1E, and 1F). Our analysis revealed the co-expression of CD4, RORC, FOXP3, GATA3, TBX21, and CD8 in a single cluster, possibly representing immune cells (e.g., cluster 3) (Figures 1D, 1E, and 1F). Interestingly, Ccl23 expression is confined to cluster 2, hinting that Ccl9(Mice)/Ccl23 (Humans) is not actively produced by T cells. However, it seems to be critical to their recruitment or activation. Conversely, IL21, CCL20, STAT3, IFNγ, RORα, and IL26 which are known CD4+ Th17 cells markers, are upregulated in cluster 3. These results hint toward activation of RORγt T cells TCR associated genes by CCL9 but do not exclude a positive effect on other CD4+ T cells phenotypes such as Th1. It has been shown that within the pool of T cells, RORγt is expressed by Th17 and by Tc17 [1]. At this stage, our investigation cannot support the exclusive recruitment or activation of either type.

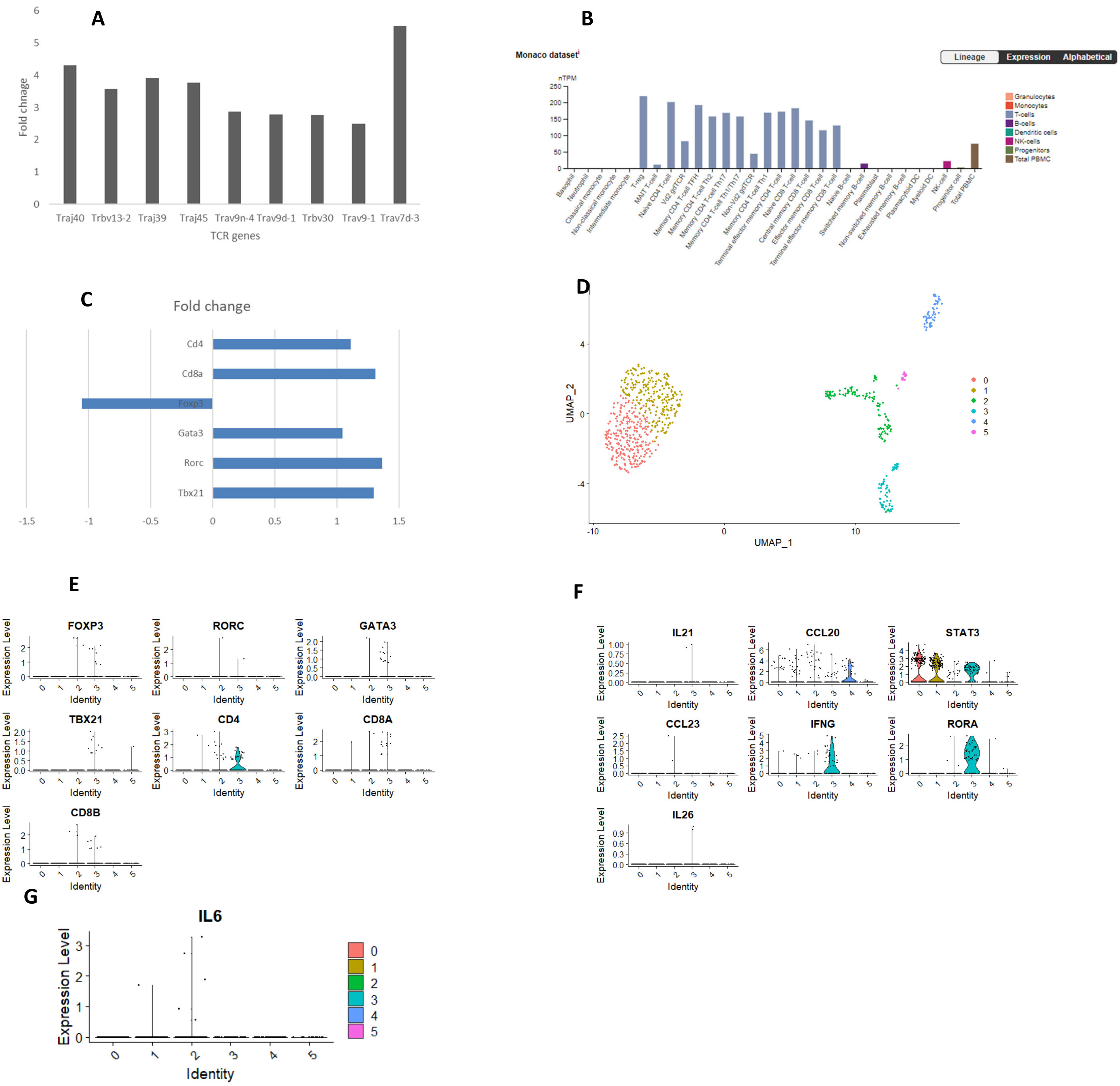

Figure S1: A bioinformatics analysis suggesting that CCL9 may play a role in the recruitment of RORγt+ T cells**.** (**A**) Our microarray analysis revealed that TRA and TRB genes, including the genes required for joining and diversifying the TCR receptor, are upregulated following

overexpression of CCL9 (**B**). Human atlas proteins levels mining confirms the upregulation of TRA and TRB-associated genes exclusively in T cells. (**C**) Primary tumors overexpressing CCL9 were associated with an upregulation of RORγt, CD4, CD8, TBX21, a slight upregulation of GATA3, and a slight downregulation of FoxP3. (**D**) scRNA-eq analysis of single cells isolated from a colorectal tumor from a patient and deposited on the GEO repository under the ID GSE222300. We clustered single cells into five main clusters using the Seurat pipeline [2]. (**E**) Expression analysis by CD4+ T cells and CD8+ main master regulators revealed that they cluster in a single cluster (Cluster 3). (**F**) Th17 cells' main markers also aggregate in Cluster 3. (**G**) We noticed that IL6 does not appear to be expressed by the same cell type as our assumed Th17 cells cluster (cluster 2 versus cluster 3, respectively). Taken together, this data hints that overexpression of CCL9 resulted in the upregulation of various TCR-associated genes, possibly through a RORγt mediated pathway.

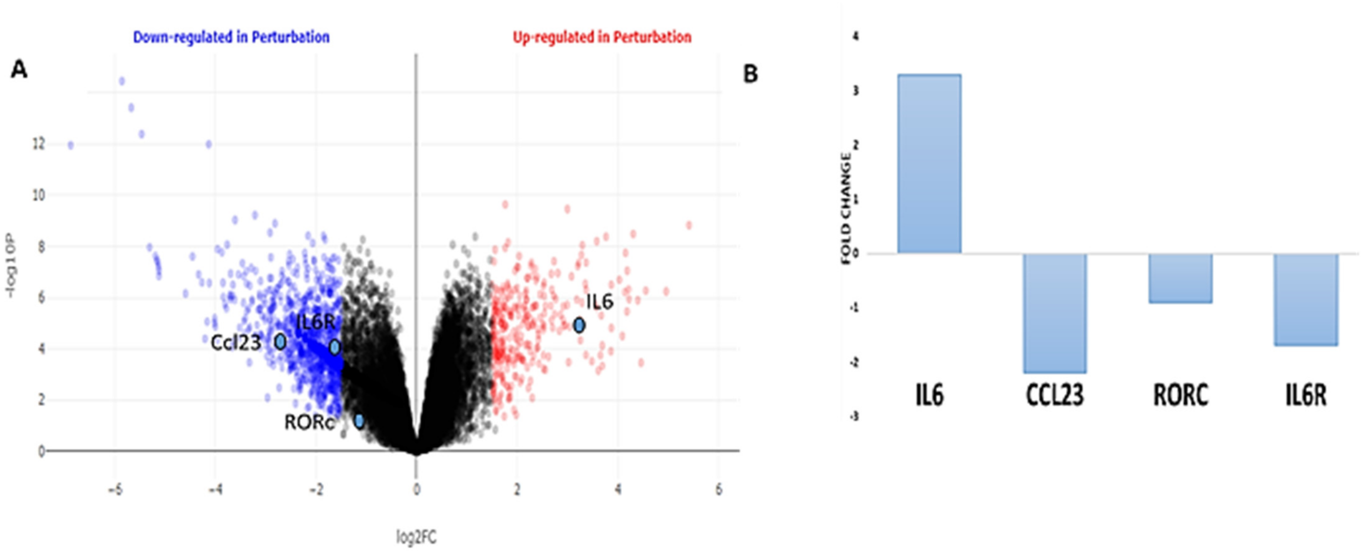

Figure S2 Bioinformatics analysis of IL6 expression in colorectal cancer. (**A**) and (**B**) Analysis of the RNA-seq data (ID: GSE50760) of 18 colorectal cancer patients shows IL6 upregulated, while CCL23 and RORc downregulated. These findings confirm that IL6 expression does not seem to follow RORγt expression cancer.

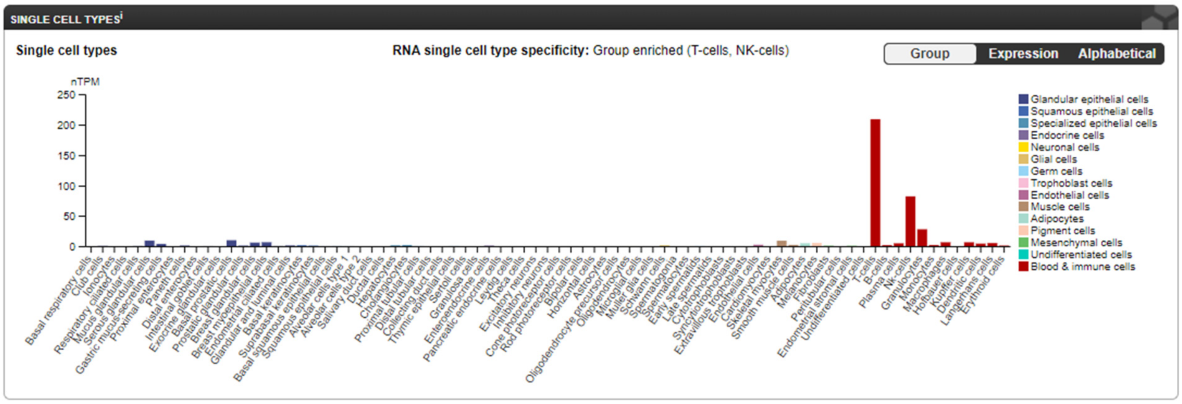

Figure S3. IFNγ expression by different cell types**.** We mined the human protein atlas for the expression of IFNγ. Our results seem to show that IFNγ is expressed by cells other than T cells, including natural killer cells and granulocytes.

References

1. Zhao , Y.; Balato, A.; Fishelevich, R.; Chapoval, A.; Mann, D.; Gaspari, A. Th17/Tc17 infiltration and associated cytokine gene expression in elicitation phase of allergic contact dermatitis. Br. J. Dermatol. **2009**, *161*, 1301–1306, https://doi.org/10.1111/j.1365-2133.2009.09400.x
2. American Thoracic Society International Conference Abstracts > B97. FLIPPING THE SWITCH: DETERMINANTS OF FIBROSIS: Adams, T.; Sakamoto, K.; Ahangari, F.; Munivar, A.; Kaminski, N. Single Cell RNA-Sequencing Reveals Distinct Effects of Inhibition of FENDRR, a Long Non-Coding RNA Implicated in Fibroblast to Myofibroblast Differentiation. *Am J Respiratory and Critical Care Medicine* 2017;195:A4664.
